# Spatial and Seasonal Structure of Bacterial Communities Within Alpine Vineyards: Trentino as a Case Study

**DOI:** 10.1007/s00248-021-01948-9

**Published:** 2022-01-13

**Authors:** Raffaele Guzzon, Daniela Bertoldi, Tomas Roman, Roberto Zanzotti, Elena Franciosi

**Affiliations:** Fondazione Edmund Mach, Via Mach 1, 38010 San Michele all’Adige, TN Italy

**Keywords:** Prokaryotic microbiota, Vineyard, Illumina MiSeq, *terroir*, Biodiversity, Training system

## Abstract

Bacteria have a fundamental role in determining the fitness of grapevine, the composition of grapes and the features of wines but at present, little information is available. In this work, the bacteria colonizing the different portions of grapevine (bark, leaves and grapes) were explored in the vineyards of the Alpine region of Trentino, considering the impact of different environmental and agronomical variables. The vineyards included in the work were selected based on their different geographical positions (altitude) and grapevine training systems in order to explore the whole variability of the grapevine ecosystem. Moreover, the surface amount of copper was measured on grapes and leaves during the vegetative growth. Bacterial analysis, performed using plate counts and Illumina MiSeq, revealed an increase in the concentration of grape bacteria proportional to the progress of the ripening stage. Conversely, the peak of bacterial concentration onto leaf and bark samples occurred in August, probably due to the more favourable environmental conditions. In bark samples, the bacterial microbiota reached the 7 log CFU/cm^2^, while 6 log UFC/g were measured in grape samples. A remarkable biodiversity was observed, with 13 phyla, 35 classes, 55 orders, 78 families and 95 genera of bacteria present. The presence of some taxa (*Alphaproteobacteria*, *Desulfovibrionaceae*, *Clostriadiales*, *Oscillospira*, *Lachnospiraceae* and *Bacteroidales*) was ubiquitous in all vineyards, but differences in terms of relative abundance were observed according to the vegetative stage, altitude of the vineyard and training system. Bacteria having oenological implication *(Lactobacillus*, *Pediococcus* and *Oenococcus*) were detected in grape samples collected in August, in low abundance. The data revealed a complex bacterial ecosystem inside the vineyard that, while maintaining common traits, evolves according to environmental and agronomical inputs. This study contributes to define the role of bacteria in the complex balance established in each vineyard between human actions and agricultural environment, known as *terroir*.

**Supplementary Information:**

The online version contains supplementary material available at 10.1007/s00248-021-01948-9.

## Introduction

Wine is not an ordinary food. Since the XII century, constant research has been conducted with the aim of improving its features and, above all, those useful to link wine to the vineyard of origin [1]. The effort of generations of winemakers has long been summarised in the idea of *terroir*. This concept includes the interaction between vines, micro-climate and agronomic choices that gives wines their distinctive characters. Historically interest focused firstly on the geology of the vineyard and the agronomic and climatic variables [2]. Oenological practices [3, 4] and microorganisms also play an important role in the definition of wine characters. The microbiota dynamic from vineyard to cellar is known to be crucial for the winemaking process [5, 6].

Few studies investigated the interactions between grapevine and bacteria in terms of plant fitness and effect on grape composition [7, 8]. Most studies on the composition and dynamics of grapevine-associated bacterial communities focused on the grape must [9–11] and bulk soil [12–14]. Only a small number of them studied bark, berry and leaf microbial communities [15–17]. It has been demonstrated that diversity and richness of bacterial species in bark were greater than in leaf and berry [16], and that bark partially shares its bacterial communities with the soil [15, 16]. By contrast, half of the genera shared by grape and leaf were not present in bark samples [17].

Climatic conditions, plant genotype, environmental factors and agronomical practices could affect the composition and abundance of vine-associated bacterial communities [18–20], but the knowledge about this topic must be incremented. The work by Martins et al. [16] was not a dynamic study, and the bacterial community was studied only in two vineyards at one-time point. The study by Vitulo et al. [17] did not include leaf tissues and considered only two time points and two vineyards.

This work reports about a descriptive investigation of the bacterial ecosystem of vineyard, considering the evolution induced by some agronomical and environmental variables. Using a meta-barcoding approach, we investigated the composition of bacterial communities of vine bark, leaf and berry, and their evolution in function of training system, stage of vegetative cycle and altitude of vineyard site. All the vineyards involved in the survey were grown according to a biological agronomical protocol. The residual presence of copper, the main pesticide allowed in biologic protocol [21], was also evaluated in relation to the agro-climatic variables, to understand its impact on the bacteria. The work focus on the prokaryotic community, because the role of yeasts in the wine ecosystem was already been well explored, and the application of high throughput non-cultural techniques recently unveiled the complexity of the bacterial ecosystem in the oenological sector that, certainly, deserves more attention. The vineyards of the Trento province (Eastern Alps, North Italy) could be a good case-study to monitor the evolution of bacterial ecosystem, because vines are cultivated at different altitudes and different training systems of grapevine coexist within the vineyards, e.g. Guyot and “Pergola” [22]. The results of this work describe in detail the complexity of the bacterial population that colonises the vineyards and its relation with the agro-environment variables, allowing conscious choices of winemakers to minimizing the environmental impact of viticulture.

## Material and Methods

### Vineyard Description and Experimental Sampling Protocol

The samples were collected from three vineyard sites in the Trento province (Eastern Alps, North Italy), located in different geographical positions in the year 2017 (Table 1). All the vineyard sites were in full production stage, with an average age of the plants between 12 and 15 years, and were cultured following a biological agronomical approach, according to the Commission Implementing Regulation (EU) No 834/2017.

**Table 1:** Main features of the six vineyard sites of sampling involved, located in the Trento province, North-eastern Alps, Italy

Location	Coordinates	Altitude (m)	Training system	Vine variety
Trento	46.13 N 11.11 E	250 (plain)	Guyot	*Pinot gris*
Trento	46.13 N 11.11 E	250 (plain)	Pergola	*Pinot gris*
Giovo	46.15 N 11.12 E	340 (hill)	Guyot	*Incrocio Manzoni*
Giovo	46.18 N 11.14 E	490 (hill)	Pergola	*Chardonnay*
Povo	46.08 N 11.17 E	600 (mountain)	Guyot	*Chardonnay*
Povo	46.08 N 11.17 E	600 (mountain)	Pergola	*Chardonnay*

Samples of trunk bark, leaf and grape berry were collected aseptically from *Vitis vinifera*. Half of the samples were analysed within 4 h for bacterial counts and half were stored at −80 °C until DNA extraction. In each vineyard site (Mountain, Hill and Plain) and for each training system (Pergola and Guyot), three sampling sites, each of about 25 m^2^, were identified in distal spatial points of different rows, excluding the five outermost rows to avoid interference with outside contaminations. In order to monitor the grapevine growth cycle, the samples were collected in six stages: Sprouting in May (MS), Sprouting in June (JS), Veraison in July (JV), Veraison in August (AV), Harvest in September (Ha) and Post-Harvest in October (PHa). At each stage of the vegetative cycle (*n*=6), for each site (*n*=3, Mountain, Hill and Plain) and for each training system (*n*=2, Pergola and Guyot), three biological replicates of leaf and bark were sampled and processed independently for bacterial counts, for a total of 108 samples of barks and leaves respectively.

For the copper analysis, a dedicated set of leaf samples was created collecting three biological replicates of leaves every 2 weeks from July to the end of September (*n*=7), for each site (*n*=3), for each training system (*n*=2) for a total of 126 samples of leaves and each sample was analysed within 24 h.

Berries were collected only when present, in the AV and Ha stages, and three biological replicates of grape bunches were sampled every 2 weeks from August to mid-September (*n*=4) for each of the vineyard site and training system (*n*=6) for a total of 72 samples. The berry grape samples were analysed both for bacterial counts and copper content.

### Quantitative Bacterial Analyses

Samples subjected to bacterial analysis were prepared by adding 180 g of sterile peptone water (0.1% w/v) to 20 g of each sample. The samples were mixed twice for 60 s at the setting “normal speed” in a laboratory blender (Stomacher Seward 3500; Worthing, West Sussex, UK), then appropriately diluted by decimal dilution using peptone water. Total aerobic bacteria (TAB) were incubated aerobically on plate count agar (PCA) at 30 °C for 2 days. Mesophilic lactic acid bacteria (LAB) were incubated under anaerobic conditions on MRS agar plates for 10 days at 30 °C. Acetic bacteria (AB) were incubated under aerobic conditions on CAAR agar plates for 7 days at 30 °C. All media had been purchased from Oxoid (Thermo Fischer, Waltham, MS). Only the bacterial colonies positive to the identification tests indicated by the International Organisation of Vine and Wine (OIV) method [23] were considered as putative AB or LAB. Results of plate counts were estimated as listed in the ISO 7218 [24].

### DNA Extraction

The DNA extracted from each of the three biological replicates was pooled for a final number of 36 barks, 36 leaves and 12 berry samples. We have six replicates of barks and leaves for each vineyard site, 18 replicates of barks and leaves for training system and 12 replicates of barks and leaves for altitude of the vineyard site. For berry grapes, we have four, six and four replicates for vineyard site, training system and vineyard altitude respectively. DNA extraction was carried out by means of FastDNA Spin Kit for Soil (MP biomedicals, Santa Ana, CA), with some modifications in the early step of DNA extraction. Briefly, in order to wash the samples and release all microorganisms from the surface, 50 g of each berry sample was placed in 100 mL of phosphate-buffered saline (PBS) at pH 7.4, and 10 g of each bark and leaf sample was placed in 20 mL of PBS. These steps were processed at 10 °C for 1 h with slow shaking and then the washing solutions were immediately processed. Two millilitres of each washing solution was centrifuged (Eppendorf 5804, Hamburg, D) for 30 min at 14,000 rpm at 4 °C, and the obtained pellet was dissolved in 2 mL of TE buffer (10 mM Tris, 1 mM EDTA, pH 8.0). DNA extraction was then carried out with the FastDNA Spin Kit for Soil following the manufacturer’s instructions with some little variations as in previous works on oenological matrix [25].

### Miseq Library Preparation and Illumina Sequencing, Illumina Data Analysis and Sequences Identification by QIIME2

The preparation of amplicon library, the quality checks and the quantification of pooled libraries were performed at the Sequencing Platform, Fondazione Edmund Mach (FEM, San Michele a/Adige, Italy). A 464-nucleotide sequence of the bacterial V3–V4 region [26, 27] of the 16S rRNA gene (*Escherichia coli* positions 341 to 805) was amplified. Unique barcodes were attached before the forward primers to facilitate the pooling and subsequent differentiation of samples. To prevent preferential sequencing of smallest amplicons, the amplicons were cleaned using the Agencourt AMPure kit (Beckman coulter) according to the manufacturer’s instructions. The DNA concentration of amplicons was determined using the Quant-iT PicoGreen dsDNA kit (Invitrogen) following the manufacturer’s instructions. In order to ensure the absence of primer dimers and to assay the purity, the generated amplicon libraries’ quality was evaluated by a Bioanalyzer 2100 (Agilent, Palo Alto, CA, USA) using the High Sensitivity DNA Kit (Agilent). Following quantitation, the cleaned amplicons were mixed and combined in equimolar ratios. Pair-end sequencing using the Illumina MiSeq system (Illumina, USA) was carried out at CIBIO (Center of Integrative Biology) – University of Trento (Trento, Italy). Raw paired-end FASTQ files were demultiplexed using idemp (https://github.com/yhwu/idemp/blob/master/idemp.cpp) and imported into Quantitative Insights into Microbial Ecology (Qiime2, version 2018.2). Sequences were quality-filtered, trimmed, de-noised, and merged using DADA2 [28]. Chimeric sequences were identified and removed via the consensus method in DADA2. Representative sequences were aligned with MAFFT and used for phylogenetic reconstruction in FastTree using plugins alignment and phylogeny [29]; Taxonomic and compositional analyses were conducted by using plugins feature-classifier (https://github.com/qiime2/q2-feature-classifier). A pre-trained Naive Bayes classifier based on the Greengenes 13_8 99% operational taxonomic unit (OTU) database (http://greengenes.secondgenome.com/), which had been previously trimmed to the V4 region of 16S rDNA, bound by the 341F/805R primer pair, was applied to paired-end sequence reads to generate taxonomy tables. The data generated by MiSeq Illumina sequencing were deposited in the NCBI Sequence Read Archive (SRA) and are available under accession number PRJNA649446 (https://www.ncbi.nlm.nih.gov/bioproject/ PRJNA649446/).

### Quantification of Copper on Leaf and Grape Berry Samples

Samples of leaves and berries for copper quantification were collected in the same vineyard sites used for bacterial analysis sampling. The sampling days were at least after 5 days after the last copper treatment. By means of a hollow punch (diameter 2.7 cm), about 100 disks were obtained from 25 leaves randomly chosen in each vineyard site, put in a plastic container with 150 ml of 1% HNO_3_ and shaken (RA 20, C. Gerhardt GmbH, Königswinter, D) for 5 min. The washing solution was analysed to determine copper content. In the same location, about 100 grape berries with pedicel, in order to avoid leaks of juice, were weighted and washed as previously detailed for leaves. Copper analysis was performed using an ICP-OES (Optima 8300, Perkin Elmer, Waltham, MA) equipped with a ceramic injector and a quartz cyclonic spray chamber. Copper was quantified at 327.395 nm using a 4-point calibration curve prepared by diluting a 1000 mg/L Cu certified solution (Merck, Darmstadt, Germany).

### Statistical Analysis

Statistical analysis was performed analyzing stage of vegetative cycle, vineyard site, vineyard altitude and training system as independent variables and the bacterial plate counts and copper concentration as dependent variables. For bacterial counts, statistical analysis (one-way Anova with post hoc Tukey HSD test) was performed on the whole set of samples (*n* = 108 for bark and leaf samples respectively and 72 for berries). For copper residues, one-way Anova with post hoc Tukey HSD test was performed on the dedicated set of samples of leaves for copper analysis (*n*=126) and on the whole set of berry samples (*n*=72).

No data transformation was applied, as data met the assumption of normality (Shapiro-Wilk W) and homoscedasticity (Levene test).

Pearson’s correlation test was used in order to determine the relations between copper concentration and total bacterial population in leaves and berries. All the tests on plate counts and copper residues were performed using the STATISTICA data analysis software system, version 9.1 (StatSoft, Inc. 2010 ww.statsoft.com).

Differences in diversity indices (OTU number and Shannon diversity index) of different samples were tested by Kruskal-Wallis test by a plug-in implemented in QIIME2. The overall structural changes of bacterial community were visualised by principal coordinate analysis (PCoA) based on both unweighted and weighted UniFrac distance matrices. The statistical significance of communities among all samples was assessed via the non-parametric PERMANOVA (permutational multivariate analysis of variance) by means of plug-in implemented in QIIME2. For the differential abundance test, the taxonomy information for each OTU sequence was provided using ANCOM method [30] by means of a plug-in implemented in QIIME2.

## Results

### Bacterial Counts in Bark, Leaf and Berry Samples in Different Stages of Vegetative Cycle

Table 2 reports the bacterial counts (TAB, putative LAB and AB) in all the stages and in different training system onto bark and leaf samples. The advancement of vegetative cycle resulted statistically significant (*p*<0.05) in driving the concentration of TAB on both bark and leaf samples. By converse, the training system was never significant in driving TAB counts (*p*>0.05), despite TAB counts observed on the samples collected from vines trained at Pergola were generally higher than in vines trained at Guyot (Table 2).

**Table 2:** Plate counts of total aerobic bacteria (TAB), putative lactic acid bacteria (LAB) and acetic bacteria (AB) counted onto barks and leaves collected in six different stages of grape vegetative cycle, from vineyards cultured with two training systems (Guyot and Pergola). Data are means ± SD of three biological replicates from each vineyard site (mountain, hill and plain; *n*=9)

Stage of vegetative cycle	Bark		Leaf	
	Pergola	Guyot	Pergola	Guyot
*TAB (log CFU/g)*				
MS	5.5 ± 0.3^a^	5.2 ± 0.2^a^	4.6 ± 0.4^a^	4.5 ± 0.5^a^
JS	6.2 ± 0.1^b^	6.0 ± 0.3^b^	4.7 ± 0.5^a^	4.9 ± 0.5^a^
JV	6.9 ± 0.1^c^	6.6 ± 0.2^b^	5.2 ± 1.2^ab^	4.9 ± 1.0^a^
AV	6.2 ± 0.1^b^	6.1 ± 0.2^b^	5.9 ± 0.3^b^	6.3 ± 0.4^bc^
Ha	5.0 ± 0.1^a^	4.8 ± 0.4^a^	5.7 ± 0.2^b^	5.6 ± 0.3^b^
PHa	6.2 ± 0.3^b^	6.3 ± 0.2^b^	6.7 ± 0.2^c^	6.6 ± 0.5^c^
*Putative LAB (log CFU/g)*				
MS	3.1 ± 0.6 ^a^	3.2 ± 0.2^a^	3.1 ± 0.6^a^	2.9 ± 0.6^a^
JS	1.2 ± 0.3^b^	1.0 ± 0.4^b^	2.8 ± 0.4^a^	2.9 ± 0.5^a^
JV	1.9 ± 0.4^bc^	1.6 ± 0.2^bc^	3.3 ± 0.2^a^	3.1 ± 1.2^a^
AV	2.1 ± 0.2^c^	2.2 ± 0.4^c^	3.8 ± 0.3^b^	3.6 ± 0.2^ab^
Ha	3.0 ± 0.1^a^	3.8 ± 0.4^a^	3.5 ± 0.3^ab^	3.6 ± 0.4^ab^
PHa	2.2 ± 0.3^c^	2.3 ± 0.2^c^	4.0 ± 0.5^b^	3.9 ± 0.3^b^
*Putative AB (log CFU/g)*				
MS	4.1 ± 0.3^a^	4.5 ± 0.3^a^	3.2 ± 0.2^a^	3.2 ± 0.1^a^
JS	4.4 ± 0.4^a^	4.9 ± 0.3^ab^	4.0 ± 0.5^b^	4.4 ± 0.6^b^
JV	5.2 ± 0.4^b^	5.4 ± 0.5^b^	4.1 ± 0.4^b^	4.1 ± 0.8^b^
AV	4.9 ± 0.3^ab^	4.9 ± 0.6^ab^	4.6 ± 0.5^b^	4.6 ± 0.5^b^
Ha	4.3 ± 0.7^a^	4.2 ± 0.6^a^	4.1 ± 0.1^b^	4.3 ± 0.3^b^
PHa	4.1 ± 0.7^a^	3.8 ± 0.6^a^	4.0 ± 0.3^b^	4.2 ± 0.4^b^

For each column, bacterial count values with a, b and c superscripts are significantly different (*p* < 0.05, one-way Anova with post hoc Tukey HSD)

Considering the Pergola training system onto bark samples (Guyot samples follow the same trend), the TAB counts increased from MS to JV stages: 6.9±0.1 log CFU/g). After the JV stage, a TAB decrease was observed during AV and Ha stages and a high recovery of bacteria during the PHa stage, above six logarithmic units (Table 2). In bark samples, presumptive AB reached the maximum concentration during AV stage (4.9±0.3 log CFU/g) while LAB peaked during the MS stage (3.1±0.6 log CFU/g).

Onto the leaf samples (Table 2), the TAB load was always at least an order of magnitude lower than in the bark samples, with a high variability among different sampling points during JS and JV stages. The TAB count in leaves reached the 5.9±0.3 log CFU/g during the AV stage; in the following stages, the leaves TAB load showed a trend similar to bark samples, with an increase during PHa stage (6.7±0.2 CFU/g). The LAB population remained always one order of magnitude below the TAB counts and the highest value was reached during the PHa stage (4.0±0.5 log CFU/g). Presumptive AB ranging around the 4 log units in all samples, without significant differences (*p*>0.05) from JS to PHa stage.

Table 3 summarises bacterial counts of grapes. TAB counts were always higher when collected from vines trained at Pergola than Guyot, but without statistical significance (*p*>0.05). The TAB counts increased up to 5 log CFU/g in the first week of September in all vineyard sites trained at Pergola and decreased by one order of magnitude at the end of September (Table 3). The TAB values in the first week of August were significantly lower than in following weeks in both Pergola and Guyot vineyards (*p* < 0.05). AB were never detected on berry samples, with the exception of bunches coming from vineyard sites located in the plain, during the 3rd week of September when AB counts were 3.4±0.3 log CFU/g. The LAB counts of bunches were always under 2 log CFU/g (*data not shown*).

**Table 3:** Total aerobic bacteria (TAB) counts on grape berries during Veraison (AV) and Harvest (Ha) in three vineyard sites of Trento province (mountain, hill and plain) cultured with two training systems (Guyot and Pergola). Data are means ± SD of three biological replicates (*n*=3)

Stage of vegetative cycle	Training system	Plain	Hill	Mountain
		*TAB (log CFU/g)*		
1st week August (AV)	Guyot	2.7 ± 0.3^a^	3.1 ± 0.3^a^	3.8 ± 0.3^a^
1st week August (AV)	Pergola	2.9 ± 0.2^a^	3.2 ± 0.2^a^	4.2 ± 0.6^a^
3rd week August (AV)	Guyot	4.5 ± 0.2^b^	4.1 ± 0.4^b^	4.7 ± 0.4^b^
3rd week August (AV)	Pergola	6.0 ± 1.2^bc^	4.0 ± 0.2^b^	4. 5± 0.2^ab^
1st week September (Ha)	Guyot	4.4 ± 0.8^b^	4.2 ± 0.5^b^	5.0 ± 0.3^b^
1st week September (Ha)	Pergola	5.6 ± 1.2^bc^	5.3 ± 0.8^c^	5.1 ± 0.4^b^
3rd week September (Ha)	Guyot	3.9 ± 0.2^ab^	3.9 ± 0.2^b^	3.7 ± 0.3^a^
3rd week September (Ha)	Pergola	4.1 ± 0.3^b^	3.9 ±0.3^b^	4.0 ± 0.2^a^

For a given column, bacterial count values with a, b and c superscripts are significantly different (*p* < 0.05, one-way Anova with post hoc Tukey HSD)

### Copper Residue Distribution in Function of Agro-climatic Variables

Table 4 reports the amount of copper measured on leaves and grapes. Regarding leaves: one-way Anova highlighted significant differences (*p*<0.05) for training system and week of sampling. In detail, the highest amount of copper was always detected from hill leaf samples with the exception of the sample in the 3rd week of August from the vineyard trained at Guyot. Copper levels in vineyards trained to Pergola were always lower than in Guyot (often this difference was also significant, *p*<0.05) with the only exception of the sample at the 3rd week of July in mountain. Considering the differences in the time, for both hill and plain vineyards, the highest copper contents were detected in the 1st and 3rd weeks of August in the veraison stage. A similar pattern was observed also for the mountain vineyard site but with a delay of 2 weeks; in fact, the highest copper contents were detected in the 3rd weeks of August and the 1st week of September in the veraison and harvest stages.

**Table 4:** Copper content measured onto grape (mg/kg) and leaf (mg/m^2^) samples at different stages of vegetative cycle sampling times in three vineyard areas of Trento province (mountain, hill and plain) cultured with two training systems (Guyot and Pergola). Results are shown as mean ± SD of three biological replicates for each point of sampling

**Stage of vegetative cycle**	**Training system**	**Plain**	**Hill**	**Mountain**
		**Leaves Cu (mg/m**^**2**^**)**		
1st week July (JV)	Pergola	36.7 ± 6.9 b	42.2 ± 2.2 a	12.9 ± 2.0 a
1st week July (JV)	Guyot	45.7 ± 2.0 c	56.2 ± 9.7 b	23.0 ± 1.5 b
3rd week July (JV)	Pergola	28.5 ± 1.9 a	49.0 ± 5.4 ab	36.9 ± 7.6 c
3rd week July (JV)	Guyot	39.4 ± 6.1 b	57.7 ± 10.7 b	31.8 ± 2.7 c
1st week August (AV)	Pergola	38.5 ± 7.6 b	65.2 ± 1.2 c	49.5 ± 9.9 d
1st week August (AV)	Guyot	38.5 ± 7.9 b	73.4 ± 3.2 d	56.1 ± 4.1 de
3rd week August (AV)	Pergola	39.2 ± 6.2 b	75.1 ± 4.3 d	62.6 ± 3.7 e
3rd week August (AV)	Guyot	47.2 ± 2.7 c	64.2 ± 6.4 c	66.8 ± 9.9 e
1st week September (Ha)	Pergola	37.1 ± 4.9 b	64.7 ± 7.8 c	63.6 ± 4.3 e
1st week September (Ha)	Guyot	46.7 ± 6.5 c	72.1 ± 10.7 d	64.1 ± 6.7 e
3rd week September (Ha)	Pergola	28.4 ± 3.0 a	54.8 ± 16.6 b	49.9 ± 7.5 d
3rd week September (Ha)	Guyot	43.8 ± 4.6 bc	67.0 ± 8.4 c	53.7 ± 6.7 d
5th week September (Ha)	Pergola	30.6 ± 2.1 ab	56.3 ± 2.1 b	53.0 ± 5.6 d
5th week September (Ha)	Guyot	42.8 ± 3.2 bc	54.2 ± 5.6 b	56.3 ± 4.9 de
**Stage of vegetative cycle**	**Training system**	**Plain**	**Hill**	**Mountain**
		**Grape berries Cu (mg/kg)**		
1st week August (AV)	Pergola	6.4 ± 1.0	13.4 ± 0.6 b	8.4 ± 0.9 a
1st week August (AV)	Guyot	6.3 ± 0.7	9.6 ± 1.3 ab	16.2 ± 0.5 b
3rd week August (AV)	Pergola	4.0 ± 0.6	10.1 ± 0.7 b	11.1 ± 0.8 ab
3rd week August (AV)	Guyot	3.8 ± 0.4	4.9 ± 1.0 a	19.7 ± 1.6 b
1st week September (Ha)	Pergola	4.4 ± 0.4	8.0 ± 1.2 ab	9.1 ± 1.3 a
1st week September (Ha)	Guyot	4.7 ± 0.4	4.8 ± 1.3 a	13.9 ± 1.8 ab
3rd week September (Ha)	Pergola	4.8 ± 0.5	8.5 ± 1.3 ab	7.7 ± 0.3 a
3rd week September (Ha)	Guyot	4.4 ± 0.5	4.3 ± 0.2 a	12.1 ± 2.3 ab

For a given column (plain, hill and mountain), copper values with different letters are significantly different (*p* < 0.05, one-way Anova with post hoc Tukey HSD)

Regarding grapes, one-way Anova highlighted significant differences (*p*<0.05) for training system and week of sampling. The copper amount was always lower than in the leaf samples. The highest amount of copper was detected from mountain berry samples, differently from leaves (highest amount in hill samples), with the only two exceptions of the 1st week of August and the 3rd week of September in the Pergola training system when the highest values were recorded from hill samples. Copper levels in vineyards trained to Pergola were always lower than in Guyot in mountain and by converse in hill samples, the copper levels were always higher in Pergola than Guyot (often these differences were also significant, *p*<0.05). No significant difference was observed for the training system in grape berries cultivated in plain vineyard sites. Considering the differences in the time, similarly to leaf samples, for both hill and plain vineyards, the highest copper contents, in berry samples, were detected in the 1st week of August. For the mountain vineyards, the delay in the veraison stage resulted in a shift of the copper peak, reached in the 3rd week of August.

Comparing copper residues with both berry and leaf TAB plate counts, no significant correlation (*p* always higher than 0.7) and no linear trend (*R*^2^ close to 0 in both leaves and berries) were evident that indicates a total absence of proportionality.

### Characteristics of the Sequencing Data and Richness in Bacterial Communities

With the exception of one bark (isolated from hill in Ha stage) and two leaf samples (isolated from Mountain in MS stage and from plain in PHa stage), the pools were successfully amplified in the bacterial V3–V4 16S rRNA gene region. A total of 3,295,512 paired-end sequences (an average of 40,685 reads per sample) were obtained (Table S1). The taxonomy classification made it possible to identify 13 phyla, 35 classes, 55 orders, 78 families and 95 genera. To address the hypothesis that species richness and biodiversity vary with sample source (grape berry, leaf or bark), geographical, environmental variables (vineyard site and stage of vegetative cycle) and/or training system (Pergola or Guyot), the intragroup diversity estimation (alpha diversity) was calculated, using the number of OTUs and the Shannon diversity index (Table 5). Both the Shannon index and the number of OTUs were significantly different between grape berry, bark and leaf samples (Kruskal Wallis, *p*<0.001). A significantly lower bacterial biodiversity was observed in berries, in comparison to leaves and barks. The most biodiverse stage was the Post-Harvest, while the effect of training systems and geographical positions was not statistically significant.

**Table 5:** OTU number (OTU) and Shannon diversity index (Shannon div. index) at different vineyard altitude, training and stage of vegetative cycle. Results are shown as mean ± SD

	OTUs	Shannon div. index
**Source of sampling**		
Bark	259 ± 72^a^	7.5 ± 0.39^a^
Leaf	273 ± 83^a^	7.5 ± 0.52^a^
Berry	180 ± 73^b^	6.7 ± 0.74^b^
**Stage of vegetative cycle**		
MS	199 ± 40^a^	7.2 ± 0.28^a^
JS	216 ± 61^a^	7.2 ± 0.56^a^
JV	257 ± 48^a^	7.5 ± 0.29^a^
AV	255 ± 80^a^	7.4 ± 0.65^a^
Ha	239 ± 75^a^	7.3 ± 0.69^a^
PHa	356 ± 96^b^	8.0 ± 0.47^b^
**Training system**		
Pergola	246 ± 83^a^	7.4 ± 0.62^a^
Guyot	260 ± 82^a^	7.4 ± 0.53^a^
**Vineyard altitude**		
Plain	261 ± 81^a^	7.5 ± 0.47^a^
Hill	246 ± 88^a^	7.3 ± 0.70^a^
Mountain	250 ± 83^a^	7.4 ± 0.61^a^

For a given column, OTU number and Shannon diversity index values with different letter superscripts are significantly different (*p* < 0.05)

### Diversity Analysis of the Bacterial Community on Barks, Leaves and Grape Berries

**Fig. 1:**
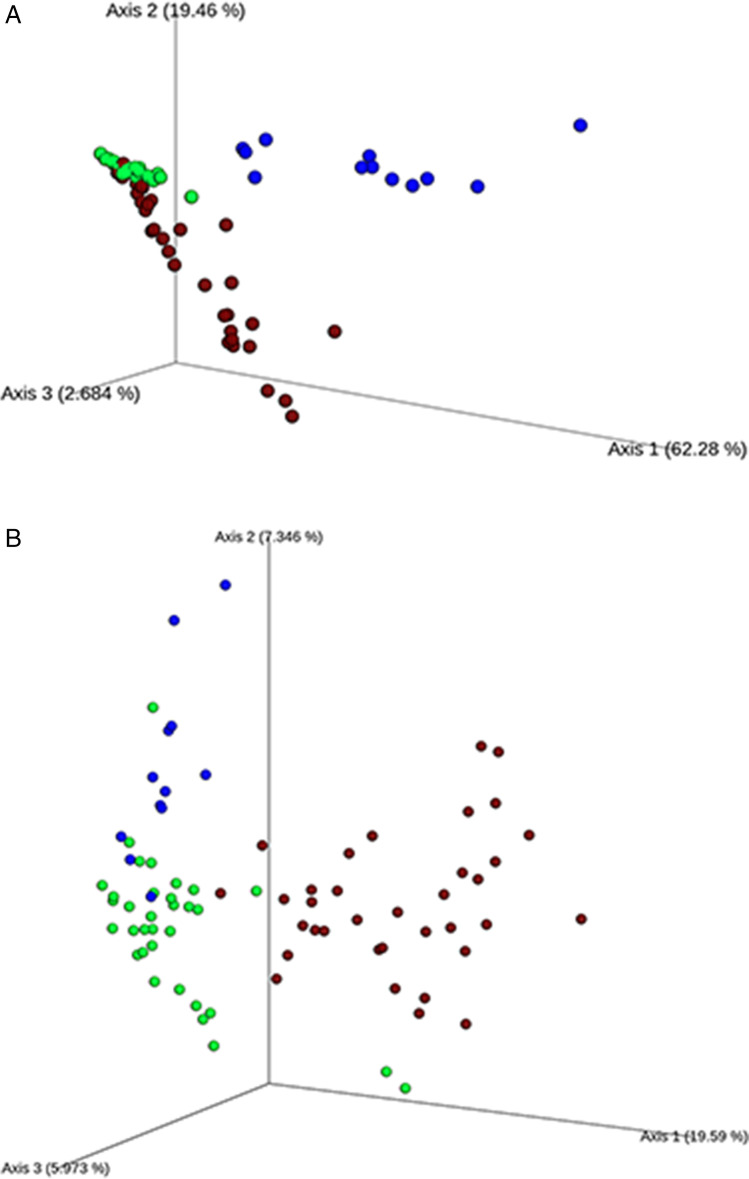
Principal coordinate analysis of weighted (A) and unweighted (B) UniFrac distances for bacterial community. Different colours represent the different sampling source: bark (brown), berry (blue) and leaf (green)

In order to assess the amount of variation in bacteria composition among the samples, we calculated the phylogenetic beta-diversity based on both weighted and unweighted UniFrac distances. The PCoA plots (Fig. 1A and B) show a clear separation of the bacterial populations between barks, leaves and grape berries, visible on both the axis 1, explaining 62% of the total variation, and the axis 2, explaining 19% of the total variation onto the weighted UniFrac PCoA plot in Fig. 1A (20% and 7.4%, respectively, onto the unweighted UniFrac PCoA plot in Fig. 1B). Since the unweighted UniFrac distance accounts for the presence/absence of OTUs, whereas the weighted type accounts for abundance too, the similar results of these two analyses suggested that the bacterial composition more than the individual bacterial abundance drives the distance among bark, leaf and berry samples.

PERMANOVA analysis explores the effects and significance of the variable sample source (bark, leaf or grape berry), vineyard site of sampling, training system (Pergola and Guyot) and stage of vegetative cycle. The test (Table 6) revealed that sample source and stage of sampling significantly affected bacterial communities (*p*<0.05). As berry, bark and leaf bacterial communities clearly segregate, as testified by PCoA (Fig. 1A and B), and showed the highest effect (the highest pseudo-*F* value in Table 6), the three data sets were analysed independently. At first, using PERMANOVA analysis, we tested the effect of the variables on the bark, leaf and berry bacterial communities. The analysis showed that vineyard and geographical position were never significant (*p*<0.05), while the sampling stage was confirmed as the principal factor affecting the bacterial population diversity of all samples. In particular, stages JS and JV showed a significant effect on both bark and leaf bacterial composition, with a higher effect in bark than in leaf samples (pseudo-*F* values higher in bark samples, Table 6). The training system was also significant in bark samples (*p*<0.05).

**Table 6:** PERMANOVA analysis (999 permutations) results for bacterial communities based on weighted UniFrac distances

**Main effects**	**Pseudo-*F***	***p*-value**
Source of sampling	48.034	0.001**
Stage of vegetative cycle	3.75	0.002**
Training system	0.653	0.53
Vineyard Altitude	0.476	0.80
**Pairwise comparisons in bark**	**Pseudo-*F***	***p*-value**
*MS vs. JS*	0.929	0.55
*MS vs. JV*	1.198	0.28
*MS vs. AV*	8.967	0.006**
*MS vs. Ha*	3.786	0.042*
*MS vs. PHa*	7.798	0.005**
*JS vs. JV*	1.634	0.29
*JS vs. AV*	15.772	0.003**
*JS vs. Ha*	5.614	0.008**
*JS vs. PHa*	11.552	0.004**
*JV vs AV*	3.431	0.054
*JV vs. Ha*	1.621	0.17
*JV vs. PHa*	3.819	0.046*
*LV vs. Ha*	1.347	0.85
*LV vs. PHa*	0.628	0.57
*Ha vs. PHa*	0.560	0.65
*Pergola vs. Guyot*	4.748	0.021*
**Pairwise comparisons in leaf**	**Pseudo-*F***	***p*-value**
*MS vs. JS*	0.774	0.66
*MS vs. JV*	3.216	0.011*
*MS vs. AV*	1.346	0.15
*MS vs. Ha*	1.609	0.064
*MS vs. PHa*	2.895	0.025*
*JS vs. JV*	3.544	0.011*
*JS vs. AV*	1.948	0.029*
*JS vs. Ha*	1.360	0.16
*JS vs. PHa*	3.432	0.018*
*JV vs. LV*	2.115	0.035*
*JV vs. Ha*	2.040	0.080
*AV vs. PHa*	3.143	0.011^*^
*AV vs. Ha*	1.347	0.18
*AV vs. PHa*	0.916	0.50
*Ha vs. PHa*	2.089	0.049*
*Pergola vs. Guyot*	1.311	0.20
**Pairwise comparisons in berry**	**Pseudo-*F***	***p*-value**
*AV vs. Ha*	6.182	0.005**
*Pergola vs. Guyot*	1.066	0.35

Significance levels: *p < 0.05; **p-value < 0.01

### Bacterial Community Structure and Differential Abundance Analysis

**Fig. 2:**
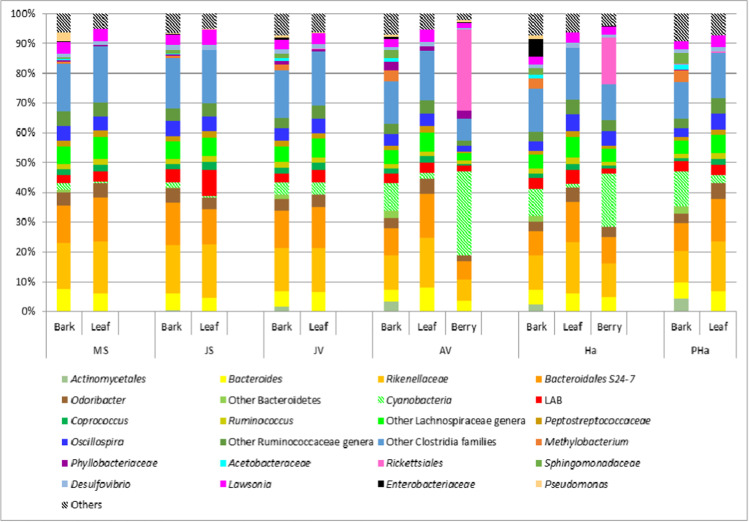
Bar graph representing the 24 dominant bacterial phylotypes (genus level or above) found across all samples of bark, leaf and berry using Illumina MiSeq. Each bar is a pool of six samples collected in different three different vineyard sites and with two different training systems (Pergola and Guyot), at six stages (MS, May Sprouting; JS, June Sprouting; JV, July Veraison; AV, August Veraison; Ha, harvesting; PHa, post-harvesting). (For interpretation of the references to colour in this figure legend, the reader is referred to the web version of this article)

Of the 24 dominant bacterial phylotypes found across all samples (Fig. 2), eight belonged to *Firmicutes*. *Lachnospiraceae* (*Coprococcus*, *Ruminococcus* and other genera) was the most abundant family of the *Firmicutes* and reached its peak during sprouting, in leaf samples. Other frequently sequenced families included *Peptostreptococcaceae*, *Clostridia* and unclassified genera belonging to the *Ruminococcaceae* family. Of the *Proteobacteria*, the genera *Lawsonia* and *Pseudomonas* were more abundant in all samples during the sprouting stages (MS and JS); *Phyllobacteriaceae* prevailed during the veraison stages (JV and AV) in bark samples, and *Rickettsiales* were predominant in berry samples. Finally, after the sprouting stages in May and June, *Methylobacterium*, *Methylocystaceae* and *Sphingomonadaceae* were more abundant. The *Bacteroidetes* constituted another dominant phylum detected in all the samples (Fig. 2) present until the JV stage in the range of 37.5–43.1%. *Bacteroidetes* abundance was always constant in leaf samples, but decreased, in both bark and berry samples after the AV stage, to a range of 18.9–28.5%. The most abundant phylotypes belonging to this phylum were the *Rikenellaceae* genus, the *Odoribacter* genus and the *Bacteroidales* S24-7 family. Other *Bacteroidetes* taxa, mainly constituted by *Chitinophagaceae* and *Cytophagaceae* families (data not shown), colonised only bark samples after sprouting in the range of 1.4–2.7%. The *Actinobacteria* phylum was totally constituted by OTUs belonging to *Actinomycetales*, a dominant phylotype present only in bark samples (Fig. 2) after the sprouting stage, and was found in the range of 1.7–4.3%. *Actinomycetales* abundance in both leaf and berry samples was never over 0.1%. The most abundant families belonging to this order were *Geodermatophilaceae*, *Microbacteriaceae*, *Nocardioidaceae* and *Sporichthyaceae*. *Archaea* and other bacterial phyla such as *Acidobacteria*, *Armatimonadetes*, *Chloroflexi*, *Fusobacteria*, *Gemmatimonadates*, *Lentisphaerae*, *Planctomycetes*, *Tenericutes*, *Deinococcus-Thermus* and *Verrucomicrobia* have always been found at very low relative abundances (never higher than 1.0%; others in Fig. 2). With regard to bacterial groups having oenological interest, LAB (Fig. 2) resulted ubiquitously distributed in all samples (bark, leaves and berry), and their relative abundances were between 1.9 and 7.3%. *Acetobacteriaceae*, including the *Gluconobacter* and *Acetobacter* genera, were detected only in bark samples and with a relative abundance never over 2%.

In order to identify taxonomic groups driving differences among the bacterial community, a differential abundance test using the ANCOM method was performed on data stratified by sample source. Some taxa were found to be differentially abundant between the stages and between agronomic training, respectively. *Phyllobacteriaceae* were more abundant in the bark samples collected during the veraison stages of July and August (JV and AV, Fig. 2); *Actinomycetales*, *Cytophagaceae* and *Chitinophagaceae* were present only in bark samples after the sprouting stages, while *Sphingomonadaceae* were more abundant in bark samples collected from vineyard trained at Pergola. No significant difference was found in terms of the vineyard site. Within the different samples data-set (bark, leaf and berry), one order (*Actinomycetales*) and seven families (*Nocardioidaceae*, *Cytophagaceae*, *Chitinophagaceae Metylobacteriaceae*, *Metylocistaceae*, *Acetobacteraceae* and *Sphingomonadaceae*) turned out to be important for characterizing bark samples; the family *Rickettsiales* characterised berry samples, whereas no taxa proved to be informative in leaf samples.

## Discussion

Considering vineyard phyllosphere-associated bacteria, this is one of the first studies that takes into account different stages of the vegetative vine cycle (sprouting in May and June, veraison in July and August, harvest in September and post-harvest in October) and is based on both quantitative methods (plate counts) and extended 16S-amplicon relative abundance description. Previous studies either reported data on bacterial communities obtained with other techniques, such as T-RFLP [16], or investigated fungal communities [31], or were based only on Illumina two-stage data analysis, without considering leaf [14, 17] or bark tissues [15]. The study of bacteria evolution thought the vegetative cycle of vine could be important to understand the effect of agro-climatic variables onto bacteria, helping to prevent vine disease and improving instrument to safety manage grape ripening, in particular in biologic agriculture, when the tools to counteract vine disease are limited both in quantitative and qualitative terms [32].

In general, our results are in agree with previous studies, since grapevine bark and leaf tissues showed significantly higher species richness than fruit berries, as previously observed for both bacteria [16] and fungi [31]. Plate counts gave a first picture of the bacterial dynamics in the vineyard. Both the position of the vineyard site and the training system did not influence TAB counts; they showed significant changes only in relation to the stage of the vegetative cycle, in agree with those observed in previous works [7, 32]. This trend was evident onto bark, which is a perennial portion of the vine, while onto leaves and berries, which are deciduous tissues, there were differences related to the geographical position of the vineyard site, probably because the location, in particular the altitude, could affect the different degree of maturity and the consequent availability of substrates for bacterial growth [7, 8]. The low presence of oenological bacteria (LAB and AB) on leaf and berry samples agrees with previous studies that recognised these microorganisms as poorly adapted to live away from wines, but it certainly questions the link between vineyard bacterial microbiota and oenological fermentation [7].

Among the many factors, including agricultural practice and geographical position, the application of agrochemicals is one of the most likely to impact the size and composition of the microbial community on leaf and berry surfaces. Our study focused on copper-based fungicides used as a common phytosanitary treatment in all vineyards. Copper is the main agent involved in contrasting *Plasmopara viticola*, and its effectiveness against the parasite is remarkable, but there are considerable issues about a possible effect on the bacterial ecosystem [21]. In our data, the amount of external copper did not influence the total bacterial load on berries and leaves. However, in leaves, a significant lower amounts of copper were detected in Pergola and this could explain the significant higher TAB concentrations observed in Pergola than in Guyot samples; in addition, the PHa stage could be considered a kind of negative control for copper content because no copper treatment was applied during PHa stage and this could explain the significantly higher TAB and LAB counts in leaves sampled in the post-harvest stage. In berries, the copper level was always lower than 20 mg/kg according to the European set limit (Reg. (EC) No 149/2008 (copper limit for grape is 50 mg/kg)). These low copper contents were probably enough to inhibit the growth of *Acetobacteriaceae* and LAB on the berry surface as already observed Verginer et al. [33], who suggested that very low abundances of microorganisms in grape samples were explained by the fact that the vineyard had been treated with CuSO_4_.

Looking at the bacteria biodiversity (alpha-diversity of the Illumina data), the data showed no difference in species richness associated to training system, geographical location of vineyard site and stage of vegetative cycle (considering from sprouting to harvest), so, if present some key bacterial-derived component for the wine quality, the considered variables will not affect them. The PHa samples of both bark and leaves showed significantly higher bacterial species richness and this could be due to the absence of copper treatment at this stage of the vegetative cycle confirming the speculation that the copper treatment could lower both amount than taxa richness of bacteria. Many other studies have mentioned exposure to copper as the cause of distinct changes in microbial community composition and harmful to biodiversity conservation in agro-ecosystems [34, 35].

All the minority bacteria, i.e. *Archaea*, *Acidobacteria*, *Armatimonadetes*, *Chloroflexi*, *Fusobacteria*, *Gemmatimonadates*, *Lentisphaerae*, *Planctomycetes*, *Tenericutes*, *Deinococcus-Thermus* and *Verrucomicrobia* were previously found in vineyard roots and soils [13, 15, 36, 37] and, with the exception of *Lentisphaerae*, *Fusobacteria* and *Tenericutes*, were already known as constituents of the Trentino vineyard soil bacterioma [38]. These results agree with the previous work by Martins et al. [16] that revealed similarities between vineyard phyllosphere and soil and strengthened the interest in research not only about berries but also about bark and leaf tissues as an informative habitat for evaluating vineyard bacterial biodiversity. Reads belonging to *Cyanobacteria* phyla are probably coming from amplification and sequencing of *V. vinifera* chloroplasts that originated from *Cyanobacteria* [39]. Most of these minority phyla were classified as having “absent/unknown effect” on wine fermentation [40].

The majority of bacteria belonged to *Actinobacteria*, *Bacteroidetes*, *Firmicutes* and *Proteobacteria* phyla. *Actinobacteria* were already known as one of the dominant phyla in the vineyard soils of the Trentino province [38]. In the present work, *Actinomycetales* mainly constituted this phylum and in particular, *Nocardioidaceae*, present only onto bark samples after sprouting. The *Bacteroidetes* phylum was almost absent in the vineyard soils of the Trentino province [38], but had been previously found in studies about vineyard soils [15, 37] and bark samples [17], classified as having “absent/unknown effect” on wine fermentation [40]. Similar to the *Actinomycetales*, the *Cytophagaceae* and *Chitinophagaceae* families, belonging to the *Bacteroidetes* phylum, were found present only onto bark samples after sprouting. Almost all the main *Firmicutes* bacterial phylotypes identified in this work have been already described as widespread on soil- and grapevine-associated bacteria [15, 17, 41–43], but not in Trentino, where they were considered absent [38]. The *Firmicutes* phylum is reported as constituted by relevant genera for winemaking, encompassing fermenting species [40].

The *Proteobacteria* phylotypes were already known to be dominant in the vineyard soils of the Trentino province [38] and were classified as having a “spoiling effect” on wine fermentation [40]. *Metylobacteriaceae*, *Metylocistaceae*, *Phyllobacteriaceae*, *Acetobacteraceae* and *Sphingomonadaceae* were found only in bark samples after the sprouting stages. These phylotypes were never detected, or detected in traces, during sprouting, in leaf and berry samples. Some species of *Sphingomonadaceae* could influence plant health and productivity [44], while the *Metylobacteriaceae* spp. may stimulate plant development through phytohormone production [45]. The *Sphingomonadaceae* and *Metylobacteriaceae* spp. can survive the wine fermentation process, and yet their impact on wine organoleptic properties remains unknown.

The identification onto bark, leaf and grape berry samples, of bacterial taxa also associated with the plant rhizosphere, like *Bacteroides*, *Odoribacter*, LAB, *Coprococcus*, *Ruminococcus*, *Oscillospira*, *Peptostreptococcaceae*, *Desulfovibrio* and *Lawsonia* phylotypes, suggested that soil and bark could be a reservoir of bacteria during the winter period, ready to colonise the aerial part of plants such as the leaf and grape berry surface. It was previously shown that cross-contaminations could often cause the leaves colonisation [46]. Indeed, it is noteworthy that *Actinomycetales* and *Acetobacteraceae* where never found on grape berries and leaves, even when present on bark tissues, in agree with plate counts. This could mean that the less abundant bacterial population cannot successfully transfer to the leaf and berry tissues, thus confirming previous results that berry samples were more similar to leaf than bark samples.

Analyzing bark, leaf and grape bacteria separately, the first finding was that the vegetative stage at sampling is the most important factor affecting the bacterial populations in all the tissues (bark, berries and leaves), and again the PHa stage showed always a significant effect on the bacterial community of leaves when compared to the other stages. The agronomic training system (Guyot and Pergola) seems to affect bark more strongly than leaf and berry bacterial community, in contrast with what previously proposed for fungi [31]; nevertheless, more studies on bark bacteria dynamic over the years would be necessary to clarify the resilience of its bacterial ecosystem.

## Conclusions

The results of this study show that the vineyard bacterial microbiota is complex and resilient, despite the many geographical location, the agronomic practices and treatments and the stage of vegetative cycle. This evidence partially contrasts with the concept of *terroir*, where each vineyard is considered as unique and inimitable. However, considering the characteristics of the vine, a perennial plant with a life cycle of several decades, we speculate that the bacterial ecosystem, adapted to the bark environment, is a reservoir for the annual colonisation of leaves and berry only partially influenced by the agronomic practices adopted. In particular, copper pollution did not seem to influence the bacterial presence onto leaves, but is probably the cause of very low amount of lactic acid bacteria and acetic bacteria on berries even when this metal is used following the European directives. The stage of the vegetative cycle is the main driving force of the evolution of the bacterial community, which makes the vineyard sensitive to possible climate changes. Finally, the bacteria of oenological interest represent only a small part of the total bacterial ecosystem. Further research is required to consider the contribution of bacteria coming from the vineyard on the progress of oenological fermentation and to understand the role of the several bacterial groups identified as not LAB or AB, with regard to the fitness of the vine and wine production.

## Supplementary Information

ESM 1 (DOCX 17 kb)

## Data Availability

Information about the availability of the data reported in this work are included in the “Material and Methods” section.
