# Supplementary material for: Spatial and Seasonal Structure of Bacterial Communities Within Alpine Vineyards: Trentino as a Case Study
Source: Microb Ecol. 2022 Jan 13;85(1):108–20. doi: 10.1007/s00248-021-01948-9 (PMC9849295; doi:10.1007/s00248-021-01948-9)
Supplement: Supplementary file 1 — (DOCX 17 kb) [file 248_2021_1948_MOESM1_ESM.docx]

**Table S1** Abundance in number of reads for all the samples sequenced for V3-V4 region of 16S rDNA gene. The sample ID is the same as reported in the NCBI Sequence Read Archive (SRA) under accession number PRJNA649446 (https://www.ncbi.nlm.nih.gov/bioproject/ PRJNA649446/).

| **Sample ID** | **N reads after filtering** | **Sample Source** | **Training System** | **Stage of the vegetative cycle** | **Altitude** |
| --- | --- | --- | --- | --- | --- |
| LAVIS-V3-V4-8L_S8 | 38683 | Bark | Pergola | MS | Mountain |
| LAVIS-V3-V4-9L_S9 | 30213 | Bark | Guyot | MS | Mountain |
| LAVIS-V3-V4-10L_S10 | 29438 | Bark | Pergola | MS | Hill |
| LAVIS-V3-V4-11L_S11 | 31503 | Bark | Guyot | MS | Hill |
| LAVIS-V3-V4-12L_S12 | 30438 | Bark | Pergola | MS | Plain |
| LAVIS-V3-V4-13L_S13 | 26129 | Bark | Guyot | MS | Plain |
| LAVIS-V3-V4-14L_S14 | 25771 | Leaf | Pergola | MS | Mountain |
| LAVIS-V3-V4-15L_S15 | 22145 | Leaf | Pergola | MS | Hill |
| LAVIS-V3-V4-16L_S16 | 23446 | Leaf | Guyot | MS | Hill |
| LAVIS-V3-V4-17L_S17 | 39706 | Leaf | Pergola | MS | Plain |
| LAVIS-V3-V4-18L_S18 | 44111 | Leaf | Guyot | MS | Plain |
| LAVIS-V3-V4-19L_S19 | 45744 | Bark | Pergola | JS | Mountain |
| LAVIS-V3-V4-20L_S20 | 43038 | Bark | Guyot | JS | Mountain |
| LAVIS-V3-V4-21L_S21 | 37793 | Bark | Pergola | JS | Hill |
| LAVIS-V3-V4-22L_S22 | 41956 | Bark | Guyot | JS | Hill |
| LAVIS-V3-V4-23L_S23 | 39398 | Bark | Pergola | JS | Plain |
| LAVIS-V3-V4-24L_S24 | 35504 | Bark | Guyot | JS | Plain |
| LAVIS-V3-V4-25L_S25 | 25402 | Leaf | Pergola | JS | Mountain |
| LAVIS-V3-V4-26L_S26 | 27440 | Leaf | Guyot | JS | Mountain |
| LAVIS-V3-V4-27L_S27 | 16323 | Leaf | Pergola | JS | Hill |
| LAVIS-V3-V4-28L_S28 | 40129 | Leaf | Guyot | JS | Hill |
| LAVIS-V3-V4-29L_S29 | 2602 | Leaf | Pergola | JS | Plain |
| LAVIS-V3-V4-30L_S30 | 25505 | Leaf | Guyot | JS | Plain |
| LAVIS-V3-V4-31L_S31 | 25194 | Bark | Pergola | JV | Mountain |
| LAVIS-V3-V4-32L_S32 | 23805 | Bark | Guyot | JV | Mountain |
| LAVIS-V3-V4-33L_S33 | 50844 | Bark | Pergola | JV | Hill |
| LAVIS-V3-V4-34L_S34 | 34488 | Bark | Guyot | JV | Hill |
| LAVIS-V3-V4-35L_S35 | 46604 | Bark | Pergola | JV | Plain |
| LAVIS-V3-V4-36L_S36 | 38417 | Bark | Guyot | JV | Plain |
| LAVIS-V3-V4-37L_S37 | 37781 | Leaf | Pergola | JV | Mountain |
| LAVIS-V3-V4-38L_S38 | 41284 | Leaf | Guyot | JV | Mountain |
| LAVIS-V3-V4-39L_S39 | 37798 | Leaf | Pergola | JV | Hill |
| LAVIS-V3-V4-40L_S40 | 39298 | Leaf | Guyot | JV | Hill |
| LAVIS-V3-V4-41L_S41 | 45041 | Leaf | Pergola | JV | Plain |
| LAVIS-V3-V4-42L_S42 | 43975 | Leaf | Guyot | JV | Plain |
| LAVIS-V3-V4-43L_S43 | 54030 | Bark | Pergola | AV | Mountain |
| LAVIS-V3-V4-44L_S44 | 43618 | Bark | Pergola | AV | Hill |
| LAVIS-V3-V4-45L_S45 | 45482 | Bark | Guyot | AV | Hill |
| LAVIS-V3-V4-46L_S46 | 39552 | Bark | Pergola | AV | Plain |
| LAVIS-V3-V4-47L_S47 | 46773 | Bark | Guyot | AV | Plain |
| LAVIS-V3-V4-48L_S48 | 45475 | Leaf | Pergola | AV | Mountain |
| LAVIS-V3-V4-49L_S49 | 44237 | Leaf | Guyot | AV | Mountain |
| LAVIS-V3-V4-50L_S50 | 45480 | Leaf | Pergola | AV | Hill |
| LAVIS-V3-V4-51L_S51 | 46642 | Leaf | Guyot | AV | Hill |
| LAVIS-V3-V4-52L_S52 | 60763 | Leaf | Pergola | AV | Plain |
| LAVIS-V3-V4-53L_S53 | 37957 | Leaf | Guyot | AV | Plain |
| LAVIS-V3-V4-54L_S54 | 44447 | Bark | Guyot | AV | Mountain |
| LAVIS-V3-V4-55L_S55 | 41042 | Berry | Pergola | AV | Mountain |
| LAVIS-V3-V4-56L_S56 | 36811 | Berry | Guyot | AV | Mountain |
| LAVIS-V3-V4-57L_S57 | 29992 | Berry | Pergola | AV | Hill |
| LAVIS-V3-V4-58L_S58 | 23130 | Berry | Guyot | AV | Hill |
| LAVIS-V3-V4-59L_S59 | 29586 | Berry | Pergola | AV | Plain |
| LAVIS-V3-V4-60L_S60 | 45155 | Berry | Guyot | AV | Plain |
| LAVIS-V3-V4-61L_S61 | 29829 | Bark | Pergola | Ha | Mountain |
| LAVIS-V3-V4-62L_S62 | 32623 | Bark | Guyot | Ha | Mountain |
| LAVIS-V3-V4-63L_S63 | 27633 | Bark | Guyot | Ha | Hill |
| LAVIS-V3-V4-64L_S64 | 23389 | Bark | Pergola | Ha | Plain |
| LAVIS-V3-V4-65L_S65 | 41980 | Bark | Guyot | Ha | Plain |
| LAVIS-V3-V4-66L_S66 | 54914 | Leaf | Pergola | Ha | Mountain |
| LAVIS-V3-V4-67L_S67 | 52014 | Leaf | Guyot | Ha | Mountain |
| LAVIS-V3-V4-68L_S68 | 44646 | Leaf | Pergola | Ha | Hill |
| LAVIS-V3-V4-69L_S69 | 53013 | Leaf | Guyot | Ha | Hill |
| LAVIS-V3-V4-70L_S70 | 39113 | Leaf | Pergola | Ha | Plain |
| LAVIS-V3-V4-71L_S71 | 52724 | Leaf | Guyot | Ha | Plain |
| LAVIS-V3-V4-72L_S72 | 7877 | Berry | Pergola | Ha | Mountain |
| LAVIS-V3-V4-73L_S73 | 58571 | Berry | Guyot | Ha | Mountain |
| LAVIS-V3-V4-74L_S74 | 39060 | Berry | Guyot | Ha | Hill |
| LAVIS-V3-V4-75L_S75 | 55111 | Berry | Pergola | Ha | Hill |
| LAVIS-V3-V4-76L_S76 | 47788 | Berry | Pergola | Ha | Plain |
| LAVIS-V3-V4-77L_S77 | 41023 | Berry | Guyot | Ha | Plain |
| LAVIS-V3-V4-78L_S78 | 58970 | Bark | Pergola | PHa | Mountain |
| LAVIS-V3-V4-79L_S79 | 46498 | Bark | Guyot | PHa | Mountain |
| LAVIS-V3-V4-80L_S80 | 24568 | Bark | Pergola | PHa | Hill |
| LAVIS-V3-V4-81L_S81 | 53861 | Bark | Pergola | PHa | Plain |
| LAVIS-V3-V4-82L_S82 | 79208 | Bark | Guyot | PHa | Plain |
| LAVIS-V3-V4-83L_S83 | 58614 | Bark | Guyot | PHa | Hill |
| LAVIS-V3-V4-84L_S84 | 61607 | Leaf | Pergola | PHa | Mountain |
| LAVIS-V3-V4-85L_S85 | 70478 | Leaf | Guyot | PHa | Mountain |
| LAVIS-V3-V4-86L_S86 | 63051 | Leaf | Pergola | PHa | Hill |
| LAVIS-V3-V4-87L_S87 | 70741 | Leaf | Guyot | PHa | Plain |
| LAVIS-V3-V4-88L_S88 | 59490 | Leaf | Guyot | PHa | Hill |
